# Cardiovascular Risk and Plasma N-terminal Pro-B-type Natriuretic Peptide in Adults With Resistance to Thyroid Hormone β

**DOI:** 10.1210/jendso/bvaf023

**Published:** 2025-02-11

**Authors:** Timothy M E Davis, Wendy A Davis, Carla Moran, Greta Lyons, Ellis Bryden, Krishna Chatterjee

**Affiliations:** Medical School, Fremantle Hospital, University of Western Australia, Fremantle, WA 6160, Australia; Department of Endocrinology and Diabetes, Fiona Stanley and Fremantle Hospitals, Murdoch, WA 6150, Australia; Medical School, Fremantle Hospital, University of Western Australia, Fremantle, WA 6160, Australia; Endocrine Section, Beacon Hospital, Dublin D18 AK68, Ireland; Endocrine Department, St. Vincent's University Hospital, Dublin D04 T6F4, Ireland; School of Medicine, University College, Dublin D04 C1P1, Ireland; Institute of Metabolic Science, University of Cambridge, Cambridge CB2 0QQ, UK; Department of Clinical Biochemistry, Addenbrooke’s Hospital, Cambridge CB2 0QQ, UK; Institute of Metabolic Science, University of Cambridge, Cambridge CB2 0QQ, UK

**Keywords:** resistance to thyroid hormone β, cardiovascular risk, N-terminal pro-B-type natriuretic peptide

## Abstract

**Purpose:**

People with resistance to thyroid hormone due to defective thyroid receptor β (RTHβ) exhibit adverse cardiovascular outcomes and premature mortality. Whether this reflects increased global cardiovascular disease (CVD) risk or hyperthyroxinemia-associated effects on cardiac rhythm and contractility is unknown. We determined CVD risk and plasma N-terminal pro-B-type natriuretic peptide (NT-proBNP) concentrations as a marker of reduced cardiac function in 99 individuals (mean age 41 years, 37% males) with RTHβ.

**Results:**

The mean (SD range) QRISK3 score for 82 participants was 2.0% (0.5-8.8%) vs 1.3% (0.3-5.0%) for age, sex, and ethnicity-matched healthy controls (*P* = .005). The QRISK3 heart age of RTHβ participants was 49.8 ± 14.5 years vs actual age 44.5 ± 12.4 years [difference 5.3 (95% confidence interval: 4.0, 6.5) years; *P* < .001]. The mean (SD range) plasma NT-proBNP in 79 RTHβ participants was 51 (18-142) pg/mL; 10.1% of values were above the age-specific 97.5th percentile of a large control sample. In multiple linear regression, age and female sex were significant independent predictors of NT-proBNP (*P* ≤ .001), but free T3, free T4, TSH, and QRISK3 10-year CVD risk were not.

**Conclusion:**

Elevated NT-proBNP concentrations, seen even in young people with RTHβ, suggest that myocardial dysfunction contributes to early adverse cardiovascular outcomes in this disorder, with increased atherosclerotic disease risk likely manifesting later in life. Measurement of NT-proBNP and assessment of cardiovascular risk should be considered at first presentation and periodically during follow-up of RTHβ.

Resistance to thyroid hormone β (RTHβ) is usually due to heterozygous mutations in the thyroid hormone receptor β (TRβ) gene [1]. It is characterized by elevated, circulating thyroid hormones with nonsuppressed TSH, dyslipidemia, and hepatic steatosis [2, 3], reflecting refractoriness to hormone action within the hypothalamic-pituitary-thyroid axis and liver, which are known to express mainly TRβ. Conversely, the action of elevated thyroid hormones via normal TRα results in hyperthyroidism of TRα-expressing tissues, including the myocardium [4].

A recent observational data linkage study showed that individuals with RTHβ were at increased risk of cardiovascular disease (CVD) morbidity and reduced survival compared with age- and sex-matched controls from the general Welsh population [5]. The median age of any first major adverse event [all-cause mortality, acute myocardial infarction, stroke, heart failure, or atrial fibrillation (AF)] was 11 years younger in the RTHβ group. While the small RTHβ sample size (n = 55) complicated the analysis of group-specific differences in individual endpoints, the authors favored deleterious effects of chronic elevations of thyroid hormones on cardiac rhythm and contractility rather than increased atherosclerosis risk from associated dyslipidemia and insulin resistance as the underlying cause of the relatively poor prognosis [5].

Although there is significant phenotypic variability, RTHβ can be associated with dyslipidemia (raised serum low-density lipoprotein-cholesterol and triglycerides and reduced high-density lipoprotein-cholesterol), hypertension, and systemic insulin insensitivity [3, 6, 7]. The resulting potential increase in global CVD risk has not been evaluated, but it can be readily assessed using equations that predict future atherosclerotic events. Among established risk scores, the QRISK3 is the most widely used in the United Kingdom [8], and it has the greatest value in younger and nonmultimorbid populations [9-11], which would typically include individuals with RTHβ.

While, as with cardiometabolic variables, there is significant interindividual variability in echocardiographic findings [7, 12], RTHβ can be associated with impaired systolic and diastolic function and dysrhythmias such as AF [13]. Plasma N-terminal pro-B-type natriuretic peptide (NT-proBNP) concentrations can identify reduced left ventricular systolic function [14, 15] but have not been evaluated in people with RTHβ. Although a raised plasma NT-proBNP was documented in a young boy with homozygous RTHβ and a dilated cardiomyopathy [16], thyrotoxicosis per se can be associated with increased plasma NT-proBNP concentrations, regardless of whether or not heart failure is present [17]. An elevated NT-proBNP in conventional hyperthyroidism is thought to reflect a variety of mechanisms including enhanced *BNP* gene transcription, direct effects on cardiomyocyte function, increased β-adrenergic activity, and a hyperdynamic circulation [17]. A study in which plasma NT-proBNP was measured in RTHβ subjects would need to take this phenomenon into consideration.

Since mechanisms underlying the adverse cardiovascular prognosis associated with RTHβ are unknown, the aim of the present study was to quantify CVD risk and measure plasma NT-proBNP in a large, unselected cohort of adult RTHβ individuals, to determine the relative contributions of atherosclerotic cardiovascular risk and hyperthyroxinemia-associated cardiac dysfunction to the recognized adverse cardiovascular outcomes in this disorder.

## Participants and Methods

### Participants and Study Protocols

Adult patients with RTHβ, harboring diverse TRβ mutations who were referred to our center over 3 decades, were assessed. Phenotyping studies included the collection of clinical data and demographic variables, together with blood biomarkers following an overnight fast. All investigations were undertaken as part of an ethically approved protocol (RTHβ: Cambridgeshire, LREC 98/154; Thyrotoxicosis, REC 05/Q0108/117) with the informed written consent of participants.

### Biochemical Measurements

Thyroid hormones (free FT4, free FT3, TSH) were measured using a DELFIA^®^ fluoroimmunometric assay (Wallac, Milton Keynes, UK). Reference ranges for these analytes were derived from an unselected cohort of healthy adult subjects with normal thyroid function (mean ± SD age 39.1 ± 11.1 years, 41% males, and mean ± SD body mass index (BMI) 26.5 ± 13.9 kg/m^2^) [3]. Serum lipids were measured by Advia Centaur (Siemens, Germany), nonesterified fatty acids by Roche Free Fatty Acids (Roche Diagnostics, Mannheim, Germany), and insulin by Diasorin XL Liason methods, with calculation of the Homeostatic Model Assessment of Insulin Resistance from fasting plasma glucose and insulin concentrations using the following formula: Homeostatic Model Assessment of Insulin Resistance = fasting plasma glucose (mmol/L) × fasting plasma insulin (mU/L)/22.5, as described previously [18]. Plasma NT-proBNP was measured using a chemiluminescent immunometric assay (Siemens IMMULITE 2000, Siemens Healthcare Diagnostics, Tarrytown, NY, USA). In addition to the participants with RTHβ, plasma NT-proBNP concentrations were available as part of baseline screening of 52 unselected patients with newly-diagnosed thyrotoxicosis seen in our center.

### Quantification of Cardiovascular Risk

The QRISK3 score was applied to available data from each individual. The QRISK3 predicts the 10-year risk of CVD, defined as a composite outcome of coronary heart disease, ischemic stroke, or transient ischemic attack, based on a range of demographic factors, clinical diagnoses, and clinical values in people aged 25 to 84 years of age without a history of heart attack or stroke [8]. For comparison, the predicted 10-year risk of a healthy person of the same age, sex, and ethnicity with no adverse clinical conditions, a serum total cholesterol:HDL ratio of 4.0, a systolic blood pressure of 125 mmHg, and a BMI of 25 kg/m^2^ was also calculated. Relative risk was derived from these 2 values. The heart age of the individual was also calculated [19].

Eligible body weight and BMI ranges for QRISK3 are ≥40 kg and ≥20 kg/m^2^, respectively; where these conditions were not met (for 1 body weight and 4 BMIs in the present study), the patient's weight and/or BMI were set at their respective minima. QRISK3 CVD risk over 10 years was quantified down to 0.1% and classified as <0.1% below this. Similarly, heart age was calculated down to 30 years and classified as <30 years if lower.

### Statistical Analysis

The computer packages IBM SPSS Statistics 25 (IBM Corporation, Armonk, NY, USA) and StataSE 15 (StataCorp LP, College Station, TX, USA) were used for statistical analysis. Data are presented as proportions, mean ± SD, geometric mean (SD range), or, in the case of variables that did not conform to a normal or log-normal distribution, median and interquartile range. For independent samples, 2-sample comparisons were by Fisher's exact test for proportions, Student's *t*-test for normally distributed variables, and the Mann-Whitney U-test for nonparametric variables. For multiple between-group comparisons, ANOVA was used. Nonparametric correlation was used to assess associations between variables. Multiple linear regression modeling of independent associates of log-transformed (ln) NT-proBNP included clinically plausible variables with bivariable *P* < .020 utilizing backward stepwise entry (entry *P* < .050 and removal *P* ≥ .050). Because of the restricted sample size, a 2-tailed significance level of *P* < .01 was used throughout.

## Results

### Participant Characteristics

The 99 participants included in the present study who had a QRISK3 score and/or plasma NT-proBNP assay (using contemporaneous data) were aged 15.7 to 67.8 years, and 37.4% were males. Their characteristics are summarized in Table 1. One patient had comorbid type 1 diabetes mellitus. Although 14 individuals (14.1%) were taking carbimazole or thyroxine, concentrations of free T4 and free T3 were elevated with a nonsuppressed TSH, consistent with the diagnosis of RTHβ. Of the 4.2% with a history of AF, all were in the 50- to 69-year age-group. Only 1 in 14 participants was statin-treated.

**Table 1.** Characteristics of participants with RTHβ

Variable	n	
Age (years)	99	40.7 ± 14.8
Sex (% males)	99	37.4
BMI (kg/m^2^)	99	26.6 ± 5.0
Smoking (% never/ex/current)	99	63.6/24.2/12.1
Atrial fibrillation (%)	96	4.2
Systolic blood pressure (mmHg)	88	129 ± 15
Diastolic blood pressure (mmHg)	88	76 ± 11
Antihypertensive therapy (%)	99	10.1
Total serum cholesterol (mmol/L)	96	4.9 ± 1.1
Serum HDL-cholesterol (mmol/L)	95	1.15 ± 0.31
Serum total:HDL-cholesterol ratio	95	4.5 ± 1.3
Serum LDL-cholesterol (mmol/L)	95	3.1 ± 0.9
Serum triglycerides (mmol/L)	95	1.4 (0.9-2.4)
Statin therapy (%)	99	7.1
Fasting serum glucose (mmol/L)	94	5.0 (4.6-5.2)
Fasting serum insulin (mU/L)*^a^*	96	7.1 (3.7-13.7)
HOMA-IR*^a^*	92	1.59 (0.80-3.18)
Plasma NEFA (μmol/L)	96	404 (262-623)
Serum uric acid (mmol/L)	79	0.33 ± 0.07
Plasma NT-proBNP (pg/mL)	79	51 (18-142)
Serum TSH (mU/L)*^b^*	99	2.7 (1.0-7.0)
Serum free T4 (pmol/L)*^b^*	91	32.0 (23.3-43.9)
Serum free T3 (pmol/L)*^b^*	98	11.3 (8.3-15.4)
Serum reverse T3 (nmol/L)	92	0.61 (0.36-1.04)

Data are mean ± SD, geometric mean (SD range), or median (interquartile range).

Abbreviations: BMI, body mass index; HDL, high-density lipoprotein; HOMA-IR, Homeostatic Model Assessment of Insulin Resistance; LDL, low-density lipoprotein; NEFA, nonesterified fatty acids; NT-proBNP, N-terminal pro-B-type natriuretic peptide; RTHβ, resistance to thyroid hormone β.

^*a*^Excluding participant with comorbid type 1 diabetes mellitus.

^*b*^Reference ranges: TSH 0.7-4.5 mU/L, free T4 9.7-17.3 pmol/L; free T3 3.4-7.0 pmol/L.

### QRISK3 Score

Fifteen participants who were <25 years old and 2 participants with a prior history of heart attack or stroke were excluded, leaving 82 participants eligible for analysis. All were White Caucasian with a mean ± SD age of 44.5 ± 12.4 (range 26.3-67.8) years, and 36.6% were males. The geometric mean (SD range) QRISK3 10-year CVD risk for the 82 participants was 2.0% (0.5-8.8%) (range 0.1-27.0), compared to 1.3% (0.3-5.0%) for age-, sex-, and ethnicity-matched healthy controls (*P* = .005). Since the lower limit of the QRISK3 heart age has been set as <30 years, the 9 participants with heart age <30 years were assumed to have a heart age equal to their actual age. The QRISK3 heart age of the RTHβ cohort was 49.8 ± 14.5 years compared to their actual age of 44.5 ± 12.4 years [difference 5.3 (95% confidence interval: 4.0, 6.5) years; *P* < .001].

### Plasma NT-proBNP Concentrations

**Figure 1.**
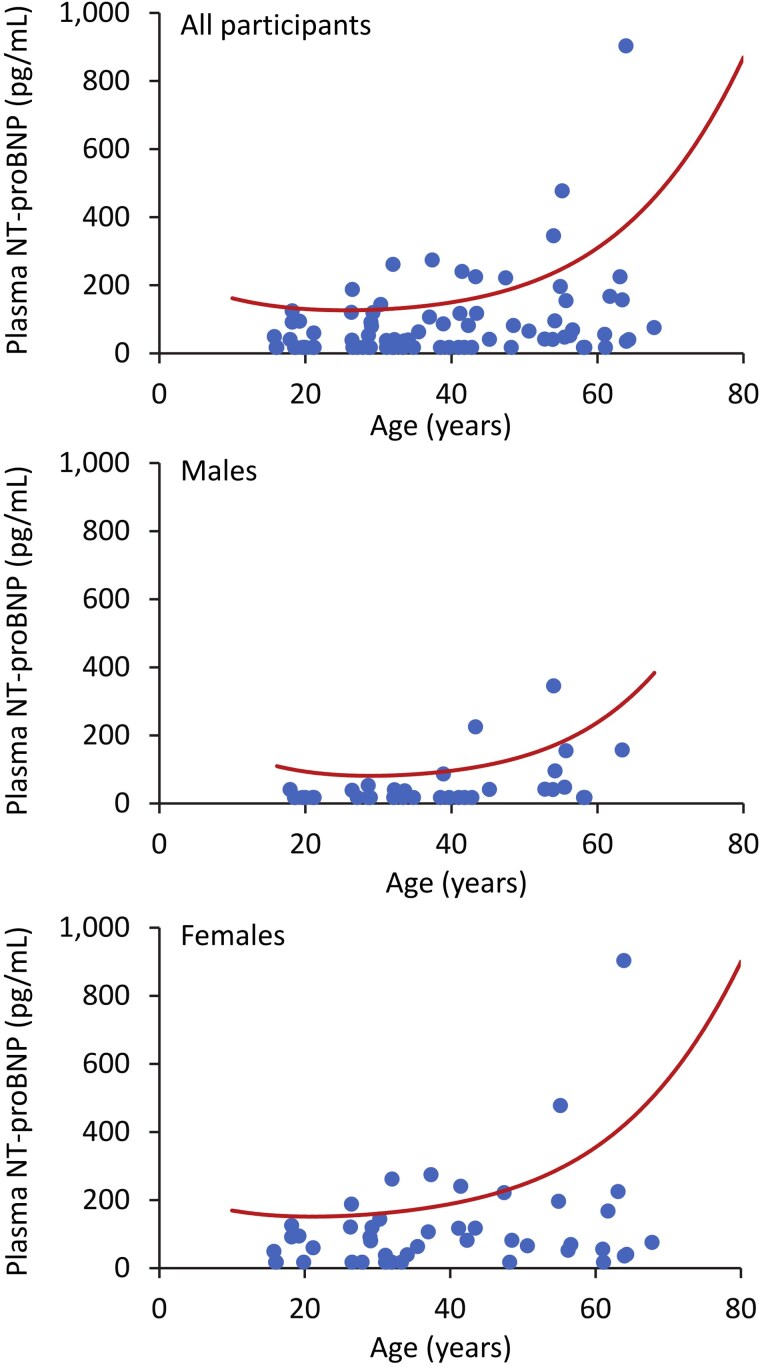
Scattergrams showing individual plasma N-terminal pro-B-type natriuretic peptide concentrations for all participants with RTHβ (upper panel) and those who were male (middle panel) and female (lower panel). The solid lines represent the 97th percentile of the normal range for non-Hispanic Whites from a large US study (20).

Of the 99 participants, valid plasma NT-proBNP concentrations were available in 79. All but 1 (South Asian) were White Caucasian with mean ± SD age 38.5 ± 14.9 (absolute range 15.7-67.8) years, and 43.0% were males. Their geometric mean plasma NT-proBNP was 51.2 pg/mL (SD range 18.5-142; absolute range 17.5-903). Individual plasma NT-proBNP concentrations plotted for the whole group are shown in Fig. 1 (upper panel). When compared with a large healthy US sample of >18 000 people [20], 8 of the 79 RTHβ participants (10.1%) exhibited plasma NT-proBNP concentrations above the 97.5th percentile for age for non-Hispanic Whites.

The bivariable associations of continuous and categorical variables with plasma NT-proBNP are shown in Tables 2 and 3, respectively. Increasing age and female sex were the only variables with a statistically significant association. In multiple linear regression, the only independent associates of ln (NT-proBNP) were age [regression coefficient (95% confidence interval) 0.023 (0.009 to 0.036) for each year increase; *P* = .001] and male sex [−0.76 (−1.16 to −0.36); *P* < .001]. Other plausible variables including free T3, free T4, and TSH, as well as QRISK3 10-year CVD risk, were not significantly associated with ln (NT-proBNP) in either bivariable analyses (*P* ≥ .076) or when included as candidate independent variables in a linear regression model. Given the significant and well-recognized associations with age and sex, the individual plasma NT-proBNP concentrations plotted for males and females are shown in Fig. 1 (middle and lower panels, respectively). In comparison with a large healthy US sample of >18 000 people [20], 2 of 34 male RTHβ participants (5.9%) and 6 of 45 female RTHβ participants (13.3%) had plasma NT-proBNP concentrations above the 97.5th percentile for age for non-Hispanic Whites. The same comparison of the RTHβ cohort with a large UK sample (also >18 000 individuals) without CVD [21] showed similar percentages (5.9% and 11.1% for males and females with a plasma NT-proBNP concentration >97.5th percentile for sequential age-groups). The participant with a NT-proBNP ≥900 pg/mL, a level associated with acute heart failure in people aged 50 to 75 years [22], was a 64-year-old female with a QRISK3 10-year CVD risk of 27.0%.

**Table 2.** Bivariable associations of continuous variables with plasma NT-proBNP

Variable	n		Nonparametric correlation coefficient (*P*-value)
Age (years)	79	38.5 ± 14.9	.319 (.004)
BMI (kg/m^2^)	79	26.8 ± 5.0	.202 (.074)
Systolic blood pressure (mmHg)	72	129 ± 15	−.065 (.586)
Diastolic blood pressure (mmHg)	72	76 ± 11	−.087 (.466)
Total serum cholesterol (mmol/L)	76	4.9 ± 1.1	.132 (.256)
Serum HDL-cholesterol (mmol/L)	75	1.08 ± 0.24	−.013 (.909)
Total:HDL-cholesterol ratio	75	4.6 ± 1.3	.040 (.733)
Serum LDL-cholesterol (ol/L)	75	3.0 ± 0.9	.075 (.524)
Serum triglycerides (mmol/L)	75	1.6 (1.0-2.5)	.062 (.598)
Fasting serum glucose (mmol/L)	76	4.9 [4.6-5.2]	−.018 (.875)
Serum insulin (mU/L)*^a^*	78	7.5 (3.8-15.1)	−.170 (.136)
HOMA-IR*^a^*	76	1.66 (0.80-3.45)	−.137 (.238)
Plasma NEFA (μmol/L)	77	410 (270-624)	.190 (.097)
Serum uric acid (mmol/L)	79	0.33 ± 0.07	−.228 (.044)
Serum TSH	70	2.7 (1.0-7.2)	.171 (.157)
Serum free T4	73	31.5 (22.9-43.4)	−.141 (.235)
Serum free T3	78	11.1 (8.1-15.3)	−.127 (.266)
Serum reverse T3	77	0.58 (0.34-1.02)	−.009 (.935)
QRISK3 score (%)	62	1.79 (0.40-7.94)	.227 (.076)

Abbreviations: BMI, body mass index; HDL, high-density lipoprotein; HOMA-IR, Homeostatic Model Assessment of Insulin Resistance; LDL, low-density lipoprotein; NEFA, nonesterified fatty acids; NT-proBNP, N-terminal pro-B-type natriuretic peptide.

^*a*^Excluding the participant with comorbid type 1 diabetes.

**Table 3.** Bivariable associations of categorical variables with plasma NT-proBNP

Variable	n (%)	Plasma NT-proBNP (pg/mL)	*P*-value
Sex (%)	79		<.001
Male	34 (43.0)	32.6 (13.8-76.9)	
Female	45 (57.0)	72.1 (26.4-196)	
Smoking status (%)	79		.063
Never	52 (65.8)	42.3 (16.3-110)	
Ex-	18 (22.8)	70.6 (26.8-186)	
Current	9 (11.4)	81.1 (22.6-291)	
Atrial fibrillation (%)	76		.028
Yes	2 (2.6)	214 (68.4-666)	
No	74 (97.4)	46.8 (18.2-120)	
Antihypertensive medication (%)	79		.847
Yes	8 (10.1)	54.7 (15.1-198)	
No	71 (89.9)	50.8 (18.8-137)	
Statin treatment (%)	79		.533
Yes	7 (8.9)	64.5 (21.1-197)	
No	72 (91.1)	50.1 (18.2-138)	
Type 1 diabetes (%)	79		n/a
Yes	0 (0)	n/a	
No	79 (100)	51.2 (18.5-142)	
Carbimazole or thyroxine therapy (%)	79		.611
Yes	10 (12.7)	59.7 (27.5-130)	
No	69 (87.3)	50.1 (17.5-143)	
History of myocardial infarction or stroke (%)	79		.043
Yes	2 (2.5)	214 (151-304)	
No	77 (97.5)	49.3 (18.1-135)	

*P*-values are derived from Student's *t*-test or ANOVA with natural logarithm of plasma NT-proBNP) as a continuous variable.

Abbreviations: NT-proBNP, N-terminal pro-B-type natriuretic peptide.

Geometric mean (SD range) plasma NT-proBNP concentrations in our 79 RTHβ participants were significantly lower than those of the 52 unselected patients with newly diagnosed conventional thyrotoxicosis [51 (18-142) vs 197 (73-530) pg/mL; *P* < .001] of similar age and sex distribution (mean ± SD age 41.7 ± 13.9 vs 38.5 ± 14.9 years, 28.8% vs 43.0 males; *P* ≥ .140).

## Discussion

We have assessed cardiovascular risk and measured plasma NT-proBNP, a biomarker of cardiac function, in a large, unselected UK cohort of adults with RTHβ. The mean QRISK3-derived 10-year CVD risk in our RTHβ cohort (2.0%) was modestly but significantly greater than in matched healthy individuals. In addition, the QRISK3 heart age of RTHβ subjects exceeded their actual age by more than 5 years. The respective risks of a plasma NT-proBNP > 97.5th percentile for age in males and females in our RTHβ cohort were 2- to 4-fold higher than expected from normal population ranges derived from 2 contemporary large-scale studies [20, 21]. Our observations suggest that the chronic effects of raised thyroid hormones on cardiac structure and function, coupled with atherosclerotic CVD at a later age, contribute to the adverse health outcomes and premature mortality previously observed in a separate RTHβ cohort from Wales [5].

In the previous publication documenting adverse health outcomes of RTHβ patients in Wales [5], Kaplan-Meier survival curves for all-cause mortality and for major adverse cardiovascular events (cardiovascular death, acute myocardial infarction, heart failure, and stroke) in individuals with RTHβ diverged from the control population at a relatively early age (around age 40 years). There was later separation (at around age 60 years) for heart failure and AF considered as individual endpoints, while acute myocardial infarction and stroke were infrequent events, occurring even later [5]. This temporal pattern suggests an excess of cardiovascular deaths associated with RTHβ occurring relatively early. In this study, we have documented a relatively modest (2%) increased QRISK3 10-year CVD risk in RTHβ, suggesting that atherosclerotic risk, due to the dyslipidemia known to be associated with this disorder, is an unlikely contributor to early adverse cardiovascular outcomes. Furthermore, we suggest that the older QRISK3 heart age exhibited by participants in our RTHβ cohort fits with their propensity to cardiovascular events at an older age [19], as observed in the recent publication from Wales [5].

Plasma NT-proBNP concentrations exceeded the 97.5th percentile for age and sex in approximately 10% of our RTHβ cohort who were aged between 15 and 67 years (see Fig. 1). The relatively acute effects of increased thyroid hormone concentrations such as increased β-adrenergic activity and a hyperdynamic circulation are known to increase plasma NT-proBNP [17]. Although several studies of conventional thyroid dysfunction have shown a significant positive correlation between free T4 and NT-proBNP concentrations [23, 24], we did not find a significant association between plasma NT-proBNP and free thyroid hormone concentrations in our RTHβ cohort. Furthermore, our observation of lower NT-proBNP levels in RTHβ than in newly diagnosed, conventional thyrotoxicosis suggests that hyperthyroxinemia of lesser magnitude but of longer (lifelong) duration in RTHβ leads to myocardial dysfunction, including nonischemic cardiomyopathic changes found on echocardiography [13, 16], which can manifest at a relatively early age in this disorder. Such changes, which can be clinically silent [25], are a substrate for an increased risk of sudden death from arrhythmias and acute heart failure [26], which, in turn, could explain the observed excess cardiovascular mortality seen in young people with RTHβ [5].

The prevalence of AF in our RTHβ cohort (4.2%) was nearly double that reported in the UK population as a whole (age 0 to >95 years; 2.5%) [27]. All AF cases were in RTHβ participants aged 50 to 69 years, consistent with the cumulative incidence of this arrhythmia in the previous, population-based outcome study from Wales [5] and at prevalence rates higher than in the general UK population of a similar age [28]. An increased risk of stroke would be expected as a consequence of a higher prevalence of AF in RTHβ, but the risk of stroke was not significantly different in individuals with RTHβ compared with matched controls in the Welsh observational study [5]. Nevertheless, the limited sample size and lower relative incidence compared with other major outcomes such as myocardial infarction and heart failure in the Welsh study [5] mean that an effect of RTHβ on cerebrovascular disease cannot be excluded.

We acknowledge that its cross-sectional nature, the relatively young age of participants (necessitating conservative assumptions for QRISK3 prediction), and associations rather than causality represent potential limitations of our study. In addition, we did not have echocardiographic data for each participant. Nevertheless, we contend that our study has important implications for the management of RTHβ. First, we recommend routine measurement of plasma NT-proBNP in RTHβ (even at a young age or in early adulthood), with raised circulating levels of this biomarker prompting echocardiographic evaluation of cardiac function. Second, we advocate periodic estimation of CVD risk in RTHβ, using the QRISK3 algorithm as a simple, inexpensive method to inform the management of cardiovascular risk factors in this disorder, especially in older patients.

## Data Availability

Some or all datasets generated during and/or analyzed during the current study are not publicly available but are available from the corresponding author on reasonable request.
